# Molecular-Level Understanding of Phase Stability in Phase-Change Nanoemulsions for Thermal Energy Storage by NMR Spectroscopy

**DOI:** 10.1021/acs.langmuir.4c02997

**Published:** 2024-09-30

**Authors:** Jungeun Park, Ulrich Scheler, Robert J. Messinger

**Affiliations:** † Department of Chemical Engineering, The City College of New York, CUNY, New York, New York 10031, United States; ‡ Center for Multi-Scale Characterization, Leibniz-Institut für Polymerforschung Dresden e.V., Dresden 01069, Germany

## Abstract

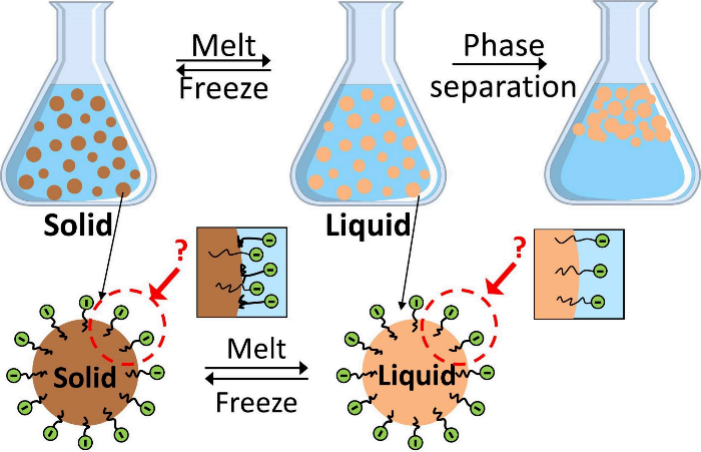

Phase change materials (PCMs) are latent heat storage materials that can store or release thermal energy while undergoing thermodynamic phase transitions. Organic PCMs can be emulsified in water in the presence of surfactants to enhance thermal conductivity and enable applications as heat transfer fluids. However, PCM nanoemulsions often become unstable during thermal cycling. To better understand the molecular origins of phase stability in PCM nanoemulsions, we designed a model PCM nanoemulsion system and studied how the molecular-level environments and dynamics of the surfactants and oil phase changed upon thermal cycling using liquid-state nuclear magnetic resonance (NMR) spectroscopy. The model system used octadecane as the oil phase, stearic acid as the surfactant, and aqueous NaOH as the continuous phase. The liquid fraction of octadecane within the nanoemulsions was quantified noninvasively during thermal cycling by liquid-state ^1^H single-pulse NMR measurements, revealing the extent of octadecane supercooling as a function of temperature. The mean droplet size of the PCM nanoemulsions, measured by dynamic light scattering (DLS), was correlated with the liquid content of octadecane to explain phase instability in the solid–liquid coexistence region. Quantitative ^13^C single-pulse NMR experiments established that the carbonyl surfactant head groups were present in multiple distinct environments during thermal cycling. After repeated thermal cycling, the ^13^C signal intensity of the carbonyl surfactant head groups decreased, indicating that the surfactant head groups lost molecular mobility. The results explain, in part, the origin of phase instability of PCM nanoemulsions upon thermal cycling.

## Introduction

Thermal energy storage is a technology that stores energy as internal energy of a material by heating or cooling.^1−4^ Such systems can bridge the gap between energy production and distribution and are used in applications ranging from solar energy plants to the thermal management of buildings.^2,3^ Internal energy can be stored within a material as sensible heat, latent heat, or a combination of them.^3^ Latent heat storage occurs when thermal energy is stored during thermodynamic phase transitions, such as melting (solid to liquid), evaporation (liquid to gas), etc. Phase change materials (PCMs) are latent heat storage materials and can absorb and release heat over narrow temperature windows associated with phase transitions. Generally, PCMs store 5–14 times more thermal energy per unit volume than sensible heat storage materials.^3^ PCMs can generally be classified as one of three types: organic, inorganic, and eutectic.^3,5^ Numerous studies have focused on organic PCMs since they exhibit a wide range of melting temperatures, which are tunable by altering their molecular structures, and are furthermore safe, inexpensive, and noncorrosive. Common organic PCMs include paraffins and fatty acids.^3,4^ However, their low thermal conductivity is a major weakness, which reduces the rate of heat transfer from PCMs to the surroundings. Another critical issue is supercooling, a phenomenon wherein the solidification of the PCM begins at a temperature well below the thermodynamic melting temperature of the oil.^1^ A wider operating temperature range decreases the energy efficiency of the energy storage system. Adding nucleating agents, such as paraffins with higher melting temperature, graphite, or hydrophobic SiO_2_, can reduce supercooling.^4^

To improve heat transfer and enable applications as pumpable heat transfer fluids, organic PCMs were emulsified in aqueous media in the presence of surfactants. PCM nanoemulsions, which have droplet diameters ranging from 10 to 500 nm,^6,7^ have high surface-to-volume ratios and thus heat exchange rates. In addition, water has a higher thermal conductivity compared to organic PCMs. Emulsification is enabled by the use of an amphiphilic surfactant that reduces the interfacial tension by adsorbing at the interface between the dispersed phase (i.e., oil) and continuous phase (i.e., water), creating electrostatic and steric barriers for coalescence. Typically, nonionic surfactants have been used, such as mixtures of Span and Tween,^4^ or anionic surfactants containing hydrophilic head groups such as carboxylate (−COO^–^), sulfate (−OSO_3_^–^), and sulfonate (SO_3_^–^).^8^

Nanoemulsions are not thermodynamically stable but can be kinetically stabilized by increasing the energy barrier for droplet coalescence.^6,9,10^ Therefore, phase instability is a critical issue for organic PCM nanoemulsions, limiting their practical applications in heat transfer systems. Creaming, flocculation, coalescence, and Ostwald ripening increase the droplet size of the emulsions over time, eventually leading to phase separation. The processes leading to phase instability can be significantly accelerated by repeated melting and freezing cycles, which can furthermore be exacerbated by shear flow during their applications as heat transfer fluids.^1,11^ To improve long-term phase stability and reduce supercooling, organic PCM nanoemulsions have been designed that contain complicated mixtures of different surfactants and cosurfactants, in combination with nucleating agents.^1,12^ For example, a surfactant mixture of polyoxyethylene sorbitan monooleate (Tween-80) and sorbitan monooleate (Span-80), in combination with graphite nanoparticles as nucleating agents, has been used to disperse paraffin in water which enhanced thermal conductivity and significantly mitigated its supercooling.^13^ Encapsulated PCMs with polymer layers have been suspended in the carrier fluid to provide large heat transfer areas, reduce PCM reactivity with the external environment, and avoid leakage or volume changes during phase change.^12^ As an example, tetradecane encapsulated by poly(methyl methacrylate) (PMMA) and polyethyl methacrylate (PEME) has been dispersed in water in the presence of surfactants, resulting in enhanced stability upon thermal cycling and improved heat transfer capabilities.^14^

To design PCM nanoemulsions with improved phase stability upon thermal cycling, it is essential to understand the molecular-level behavior of the surfactants and oil phase upon melting and freezing. However, most studies have focused on the macroscopic rheological characteristics and the phase stability of the PCM nanoemulsion. The distribution and dynamics of surfactants at the oil–water interface and how they change upon thermal cycling are poorly understood but are expected to correlate with observed rheological, momentum, and heat transfer instabilities. Liquid-state NMR spectroscopy is a powerful and noninvasive tool to study molecular structures and dynamics, while the experiments can be performed under technologically relevant conditions that reflect its environment during use.^15,16^ Via the chemical shift and NMR relaxation properties (here, dominated by stochastic, fluctuating dipole–dipole interactions), molecular-level information on environments, structures, and dynamics can be obtained.^17^

In this work, we designed a model PCM nanoemulsion system to facilitate NMR analysis using octadecane as an organic PCM oil phase, stearic acid as the surfactant, and aqueous NaOH as the continuous phase. We monitored the droplet size distribution of PCM nanoemulsions at different storage conditions over time using dynamic light scattering (DLS). To better understand the molecular origins of phase instability in PCM nanoemulsions, liquid-state ^1^H and ^13^C NMR methods were applied during thermal cycling to a PCM nanoemulsion. One-half of the stearic acid content was uniformly ^13^C-labeled, which enabled the direct, noninvasive observation of the surfactant head groups. The liquid-state NMR results were correlated with emulsion size and macroscopic phase stability, yielding insights into the molecular origin of phase instability of PCM nanoemulsions upon thermal cycling.

## Materials and Methods

### Materials

Octadecane (99%), stearic acid (98.5%), stearic acid-^13^C_18_ (99%), deuterium oxide (99.9%), 3-(trimethylsilyl)-1-propanesulfonic acid sodium salt (DSS) (97%), toluene-d_8_ (99.6%), benzene-d_6_ (99.6%), and anhydrous sodium hydroxide (NaOH) were purchased from Millipore Sigma. Deionized water was used and prepared in-house (Milli-Q). The chemical structures and physical properties of *n*-octadecane and stearic acid are shown in Figure S1a and Table S1, respectively.^18−20^

### Synthesis of PCM Nanoemulsions

Octadecane was melted in an oven at 40 °C before use. The temperature of the heating plate (CIMAREC^+^, Thermo Scientific) and ultrasonic bath (Elma ultrasonicator, 37 MHz, 90% power) were set at 75 and 70 °C, respectively. Stearic acid powder was precisely weighed in a glass vial using an electronic balance and then molten octadecane was put into the vial on the hot plate. Subsequently, the mixed solution was moved into an ultrasonic bath and kept at 70 °C for 5 min. Once the stearic acid was fully dissolved in octadecane, the solution became clear; 0.05 M NaOH aqueous solution was preheated on the heating plate at 75 °C and added to the octadecane and stearic acid mixture using a micropipette once it reached the desired temperature. The mixture was sonicated in the ultrasonic bath for 60 min at 70 °C. The emulsion was then stored at room temperature.

### Dynamic Light Scattering (DLS)

DLS measurements were performed to measure the mean droplet size (diameter) and size distributions of the PCM nanoemulsions as a function of time and temperature. The droplet size distributions were determined using a laser particle size analyzer (Zeta Sizer ZS, Malvern Instruments) either at 25 °C (for aging studies) or during thermal cycling. The analyzer has a measuring range of particles from 0.3 nm to 10 μm and a 633 nm wavelength laser as a light source. The measurement was performed at a scattering angle of 173° in polystyrene disposable cuvettes (DTS0012, Malvern Panalytical). PCM nanoemulsions were diluted to 0.1% by volume (i.e., by a factor of 1000) in deionized water, and the measurements were carried out at 25 °C with a 120 s temperature equilibration time. A non-negative least-squares algorithm was used to extract the rate of decay of the correlation function for different size classes to produce an intensity distribution.^21,22^ The intensity distribution was converted, using Mie's theory, to a volume distribution.^22^

A refractive index of octadecane was reported to be 1.439 at 25 °C, measured using a wavelength of 589 nm.^23^ The refractive index of octadecane is 1.468 for solid octadecane and 1.432 for liquid octadecane, measured using a wavelength of 600 nm.^24,25^ When the difference in wavelength is small (here, 11 nm), the effect of the wavelength on the refractive index can be neglected.^22^ Here, to calculate the volume-weighted droplet size distribution of PCM nanoemulsions, a refractive index of 1.468 was used for the solid octadecane below 15 °C, 1.439 was used for solid–liquid octadecane from 16 to 27 °C, and 1.432 was used for the liquid octadecane above 28 °C. Note that we did not take the average of the refractive index of solid and liquid octadecane as it changes nonlinearly with temperature.^25^

For DLS measurements conducted during thermal cycling, the sample was first diluted as mentioned above and cooled to 10 °C. The sample was heated to 40 °C and then cooled to 10 °C while measurements were taken in 1 °C increments. A temperature equilibration time of 2 min was used between the 1 °C increments, and the measurement duration was 5 min.

### Stability Evaluation of PCM Nanoemulsions

The emulsion stability is strongly affected by the droplet size of the emulsion.^4,26,27^ Without an increase in droplet size due to coalescence and Ostwald ripening, the phase separation of emulsions does not occur, and the emulsions are stable. Among various techniques to measure the droplet size distribution including NMR and video-enhanced or electrical microscope, DLS size measurements are the most widely used technique to measure the droplet size distribution in W/O emulsions.^26^ Visual observation of the phase separation of dispersed and continuous phases over the storage period is the easiest and simplest way to assess the destabilization of emulsion.^27^ We used the visual observation method accompanied by the DLS size measurements technique to determine the stability of PCM nanoemulsions.

PCM nanoemulsion samples were stored in airtight glass vials at ambient temperature or in an oven at 40 °C. During aging, the emulsions phase separate over time and creaming occurs due to the density difference between the water and oil phases.^1^ We monitored the time required for creaming to occur, while the size of the emulsion droplets was monitored by DLS over time. We conducted an aging experiment with two samples each aged at ambient temperature and 40 °C. The average emulsion size reflects the mean of the two samples at each temperature, while the droplet size distribution is reported from one representative sample. All samples for DLS measurements were collected from the center of PCM nanoemulsions in the vials and were taken before macroscopic phase separation occurred.

### NMR Spectroscopy

Liquid-state NMR experiments were performed using a Bruker AVANCE III HD 600 MHz NMR spectrometer equipped with a 14.1 T narrow-bore (51 mm) superconducting magnet operating at 600.14 and 150.92 MHz for ^1^H and ^13^C nuclei, respectively. A Bruker liquid-state TXI probe head was used for ^1^H and ^13^C NMR measurements, except for the ^13^C NMR measurements of PCM nanoemulsions containing ^13^C-enriched stearic acid, which were performed using a 5 mm triple-resonance inverse TCI CryoProbe. A ^1^H radiofrequency field strength of 33.4 kHz (π/2 pulse of 7.48 μs) and 27 kHz (π/2 pulse of 9.25 μs) were used for the TXI and TCI cryoprobe, respectively. A ^13^C radiofrequency field strength of 17.9 kHz (π/2 pulse of 14 μs) and 20.8 kHz (π/2 pulse of 12 μs) were used for the TXI and TCI cryoprobe, respectively.

Quantitative ^1^H single-pulse spectra were obtained with presaturation of the water signal during thermal cycling.^28^ The recycle delay of 10 s was used to ensure complete relaxation of all nuclear spins between pulses (>5 times ^1^H *T*_1_ of PCM nanoemulsions, where the *T*_1_ is the longitudinal relaxation time^29^). Quantitative ^13^C single-pulse spectra were obtained with the inverse-gated-decoupling sequence using a 30° flip angle with a recycle delay of 10 s (>5 times ^13^C *T*_1_ of PCM nanoemulsions).^30^ All ^13^C NMR measurements were acquired under ^1^H heteronuclear through-bond decoupling using the WALTZ-16 decoupling scheme. ^1^H and ^13^C *T*_1_ relaxation times were measured by using the inversion recovery pulse sequence. Temperature cycling experiments were performed by first cooling down the sample from 20 to 5 °C. Then, temperature cycling began by heating the sample from 5 to 40 °C and subsequently cooling the sample from 40 to 5 °C, where spectra were acquired at selected temperatures (see below). The temperature equilibration time between measurements was 15 min, after which no changes were observed to occur in the NMR spectra.

For all NMR experiments, fresh PCM nanoemulsion samples were prepared immediately before running experiments, and the droplet size distribution of the sample was measured using DLS to confirm that the emulsion system exhibited the expected mean droplet size and distribution. Fresh PCM nanoemulsions were inserted into a 5 mm NMR tube, while a 3 mm NMR tube containing D_2_O with 2 mM of DSS was inserted coaxially. At each temperature, the ^1^H and ^13^C CH_3_ signals of DSS were assigned to 0 ppm to remove any minor changes of chemical shift associated with temperature differences.^31^

### Extended Thermal Cycling Experiment

PCM nanoemulsion samples (20 mL) were temperature-cycled from 5 to 40 °C using an oven (ESPEC North America Inc.). One temperature cycle is defined as a heating step from 5 to 40 °C, a 90 min equilibration period at 40 °C, a cooling step from 40 to 5 °C, and a 90 min equilibration period at 5 °C. A total of 120 thermal cycles were conducted. Droplet size distributions of the samples were measured every 40 cycles by DLS. The average value of the mean droplet sizes of the three samples was obtained.

## Results and Discussion

### Finding a Model PCM Nanoemulsion

To understand the molecular behavior of surfactants in PCM nanoemulsions, we created a model PCM nanoemulsion system consisting of octadecane as the organic PCM, stearic acid as the surfactant, and water as the continuous phase. This simple model system facilitated liquid-state NMR analysis. Octadecane is one of the most studied PCMs because it melts from 26 to 29 °C, which is close to ambient conditions (Table S1).^18,20^ Octadecane and stearic acid have molecularly simple structures (Figure S1a), while ^13^C isotopically enriched stearic acids are commercially available.

To find a stable PCM nanoemulsion composition, we designed an experimental composition matrix, synthesized the samples, and compared the phase separation behavior of each sample over time at ambient temperature (Table S2 and Figure S2). To make stearic acid an anionic surfactant, NaOH was added as a base to deprotonate it. The p*K*_a_ value of stearic acid in aqueous media has been reported to range from 8.0 to 10.15^32,33^ with variations based on the measurement technique, concentration, and temperature.^34^ A sufficient quantity of NaOH should be added to increase the pH of the solution and deprotonate the stearic acid. However, if too much NaOH is added, the emulsion becomes unstable due to the screening effects of the salt.^9^ Therefore, the concentration of surfactant, oil, and salt must be balanced to form a stable PCM nanoemulsion.

The octadecane content was fixed at 20 wt %, the stearic acid content was varied from 2.5 to 10 wt %, and the pH of the aqueous phase was varied by using 0.01–0.7 M NaOH. We then observed the phase separation behavior of samples over time. The most stable PCM nanoemulsion composition contained 20 wt % octadecane, 2.5 wt % stearic acid, and 0.05 M NaOH, which will be used in this study. The pH of this model PCM nanoemulsion was measured to be 7.78–8.22 at 25 °C, which is lower than the reported p*K*_a_ values of stearic acid. This result indicates the possibility of two distinct forms of stearic acid coexisting at the oil–water interface: one in a deprotonated state and one in a protonated state. A schematic image of the model PCM nanoemulsion is shown in Figure S1b.

### Stability of a Model PCM Nanoemulsion

The stability of selected PCM nanoemulsions was investigated by (i) visually observing the macroscopic phase separation of the samples and (ii) monitoring changes in the droplet size distributions when stored at different temperatures. Samples were stored at ambient temperature or in an oven at 40 °C and photographs were taken during the storage period (Figure S3). Samples stored at ambient temperature showed better stability compared to the ones stored at 40 °C. We observed a boundary between the top milky emulsion layer and the bottom depleted emulsion layer, which is a sign of creaming in samples stored at ambient temperature after 8 weeks, while in samples stored in an oven at 40 °C after 4 weeks. The stability of emulsions could be improved, for example, by decreasing droplet size distribution and increasing the viscosity of the continuous phase.^35^ However, the stability of the model PCM nanoemulsion was sufficient to enable the NMR studies described below.

**Figure 1:**
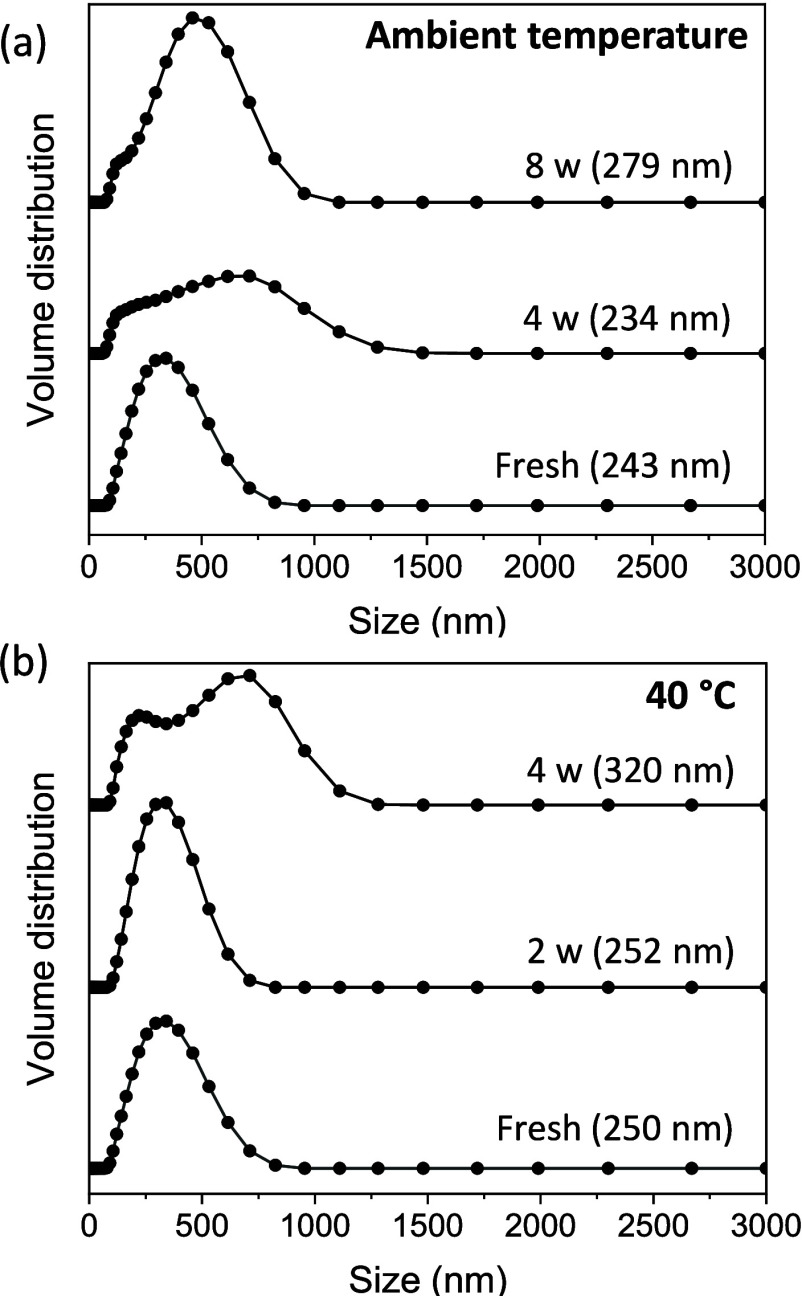
Droplet size distribution of PCM nanoemulsions stored at (a) ambient temperature or (b) 40 °C. The DLS measurement temperature was 25 °C. The aging time and average emulsion sizes are shown for each experiment, where “w” denotes the number of weeks of storage after synthesis.

The average size of emulsion droplets and the droplet size distribution of samples at different storage periods were measured by DLS at 25 °C (Figure 1) until creaming was observed, which occurred at 4 and 8 weeks at ambient temperature and 40 °C, respectively. The average droplet size increased from 243 to 279 nm at ambient temperature and 250–320 nm at 40 °C until creaming was observed. Also, the droplet size distribution became broader as the storage period increased. This trend showed that flocculation and then creaming of the emulsion droplets occurred and the emulsion became unstable during the storage period.

### Molecular Structures of Components and PCM Nanoemulsions

**Figure 2:**
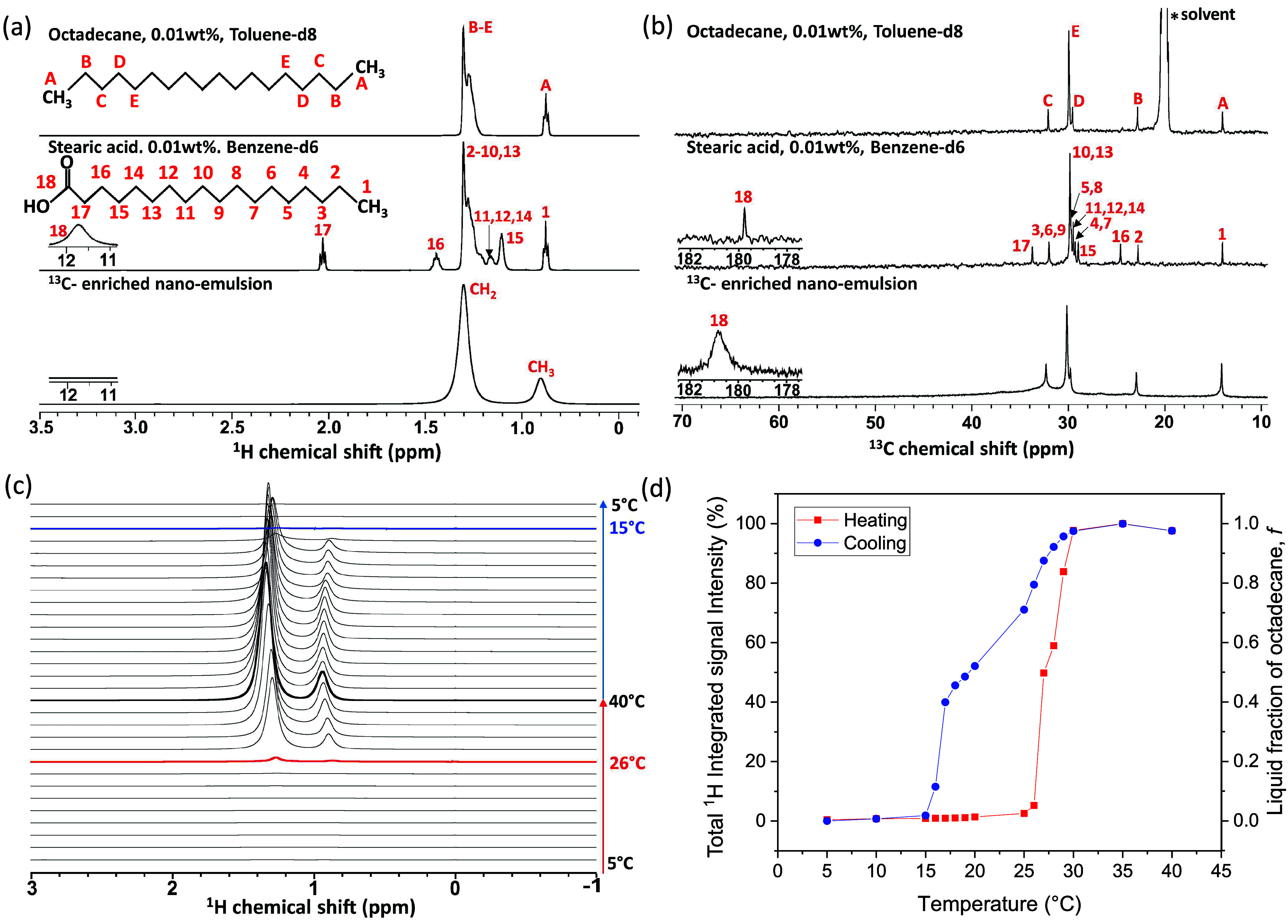
Liquid-state (a) ^1^H and (b) ^13^C single-pulse NMR spectra of octadecane, stearic acid, and ^13^C-enriched PCM nano emulsion (20 wt % octadecane, 2.5 wt % stearic acid (uniformly labeled), 77.5 wt % aqueous 0.05 M NaOH). Schematic diagrams of octadecane and stearic acid molecules are labeled with signal assignments for ^13^C moieties and their covalently bonded protons. Quantitative liquid-state (c) ^1^H single-pulse NMR spectra and (d) the integrated total ^1^H signal intensity of a model PCM nanoemulsion during thermal cycling, which yields the average liquid fraction of octadecane (*f*) within the nanoemulsions.

Liquid-state ^1^H and ^13^C NMR measurements were acquired on octadecane, stearic acid, and the model PCM nanoemulsion to assign the ^1^H and ^13^C NMR signals to the molecular structures of the various components (Figure 2a,b).^36−39^^1^H and ^13^C signal assignments of stearic acid were made using a liquid-state 2D ^1^H{^13^C} through-bond correlation NMR spectrum (Figure S4). Octadecane and stearic acid samples were prepared with 0.01 wt % of pure octadecane and pure stearic acid in deuterated toluene and benzene, respectively. To enhance ^13^C NMR sensitivity, the PCM nanoemulsion sample was synthesized using 50 mol % of ^13^C-uniformly labeled stearic acid and 50 mol % of unlabeled stearic acid. All experiments were performed at 20 °C. The PCM nanoemulsion was first melted at 40 °C and then cooled to 20 °C. In the liquid-state ^1^H NMR spectra, the ^1^H carbonyl signal of stearic acid (“18” in Figure 2a) is visible at 11.7 ppm, but not visible in the model PCM nanoemulsion as it is deprotonated. As shown below, liquid-state ^1^H NMR measurements establish that the stearic acid is deprotonated at 20 °C, in contrast to the ^13^C-labeled stearic acid carbonyl headgroup in PCM nanoemulsion, which is visible. This result may be due to rapid chemical exchange between the headgroup proton and H_2_O in the aqueous phase. In addition, the ^1^H signal at 2.03 ppm associated with −CH_2_– groups adjacent to the carbonyl signal (“17” in Figure 2a) disappear in the PCM nanoemulsion due to their reduced molecular mobilities near the oil–water interface. In the PCM nanoemulsion, the ^1^H signals of methylene (“CH_2_” in Figure 2a) and methyl (“CH_3_” in Figure 2a) groups are observed at 1.33 and 0.93 ppm, respectively. These signals mainly originate from octadecane as opposed to stearic acid, which are 20 and 2.5 wt % of the mixture, respectively, and are indistinguishable in the ^1^H NMR spectrum. In the liquid-state ^13^C NMR spectrum of the PCM nanoemulsion, however, the ^13^C signal at 181 ppm (“18” in Figure 2b) is ascribed to the carbonyl headgroup of stearic acid where the electronegative oxygen atom is double-bonded. This signal is important because it is unique to the surfactant headgroup. The ^13^C carbonyl headgroup signal of protonated stearic acid was not observed at 20 °C below the melting temperature of octadecane likely due to line broadening from the reduced molecular mobilities. Using ^13^C-uniformly labeled stearic acid, we could observe the ^13^C signal of the surfactant headgroup in the PCM nanoemulsion directly by ^13^C NMR experiment.

### Octadecane Melting and Freezing Temperature

Melting and freezing of octadecane in the PCM nanoemulsion were monitored by variable-temperature liquid-state ^1^H NMR experiments (Figure 2c). Variable-temperature ^13^C NMR experiments (Figure S5) showed a similar trend. Samples were heated from 5 to 40 °C and then cooled to 5 °C to change the phase of octadecane from solid to liquid while maintaining water in the liquid phase. Below the freezing temperature of octadecane, no ^1^H signals are observed from the solid phase. Resolved ^1^H signals from liquid octadecane appear above 26 °C as it begins to melt, as liquid-like molecular mobility begins to average out anisotropic NMR interactions (in particular, magnetic dipole–dipole couplings and chemical shift anisotropy), narrowing the line widths and enhancing sensitivity and resolution. Upon cooling, the octadecane ^1^H signals begin to decrease below 30 °C as it begins to freeze but do not disappear until 15 °C when it is completely frozen. Note that solid-state ^13^C{^1^H} cross-polarization magic-angle-spinning (CP-MAS) NMR measurements acquired from 10 to 50 °C (Figure S6) suggest conformational changes occur in octadecane upon melting, an interesting observation left for future investigations.

The total ^1^H integrated area of the alkyl signals from 0.2 to 2 ppm was normalized by the maximum ^1^H signal intensity (Figure 2d). The normalized ^1^H integrated area thus quantifies the average liquid fraction of the oil phase. We observed a hysteresis upon heating and subsequent cooling, which allowed us to observe and quantify the extent of octadecane supercooling. Here, both solid and liquid octadecane coexist within a large temperature range upon cooling (15 °C window), revealing significant supercooling. As discussed below, this coexistence region enables droplet coalescence and is detrimental to phase stability.

This result highlights the substantial benefits of utilizing liquid-state NMR spectroscopy for the nondestructive and quantitative monitoring of thermal hysteresis of PCMs selectively within the dispersed phase during thermal cycling. Differential scanning calorimetry (DSC) is currently the most widely used method to measure the degree of supercooling related to the phase transition temperature of PCM systems.^1^ However, researchers have noted that quantifying the extent and temperature range of supercooling in PCMs using DSC can be challenging to reproduce among samples, as heating rates, temperature gradients, and sample mass can affect supercooling.^40^ Compared to DSC, the temperature at each step has been shown to be equilibrated, eliminating the effects of heating rates, while no indication of temperature gradients has been observed.

### Chemical Environment of Surfactant Head and Tail Group Derived from ^13^C NMR

**Figure 3:**
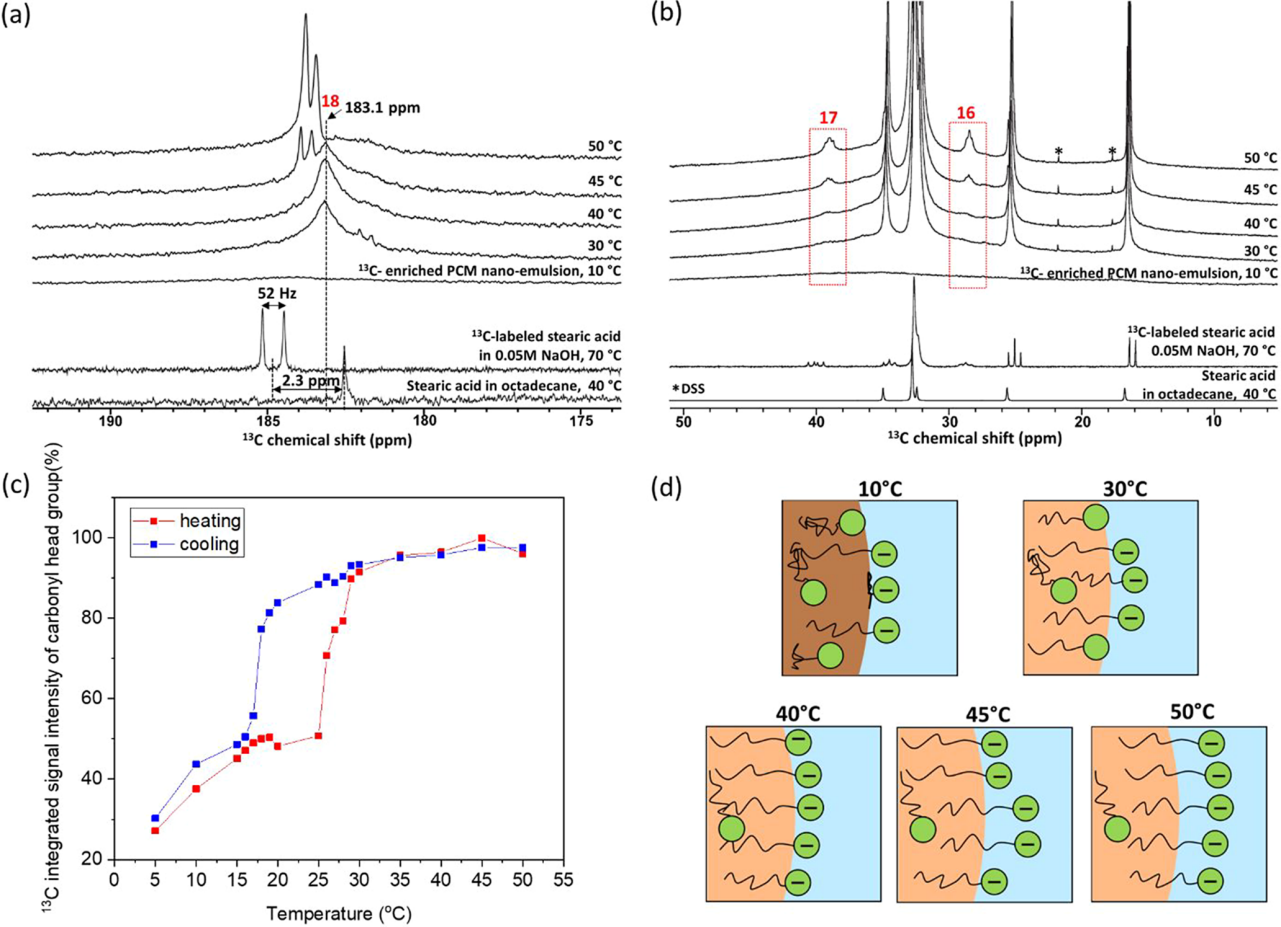
Quantitative liquid-state ^13^C single-pulse NMR spectra of (a) carbonyl headgroup moieties and (b) aliphatic tail groups of a PCM nanoemulsion (20 wt % octadecane, 2.5 wt % stearic acid (uniformly labeled), 77.5 wt % aqueous 0.05 M NaOH) acquired at different temperatures, as well as uniformly ^13^C-labeled stearic acid in 0.05 M NaOH at 70 °C and unlabeled stearic acid in octadecane at 40 °C. (c) Integrated ^13^C signal intensities of the stearic acid ^13^C carbonyl headgroup as a function of temperature. (d) Schematic images of proposed surfactant molecular behavior while heating. The blue background indicates water, and the brown and beige large circles represent frozen and melted octadecane, respectively. The green circles and black tails depict the stearic acid head and tail groups.

Variable-temperature liquid-state ^13^C single-pulse NMR experiments were acquired under quantitative conditions on the PCM nanoemulsions to measure how the molecular-level environments of the stearic acid head groups change upon octadecane phase change (Figure 3a). As above, to enhance the sensitivity of the ^13^C carbonyl headgroup, PCM nanoemulsions were synthesized with ^13^C-enriched stearic acid 50% uniformly labeled. In addition, ^13^C single-pulse NMR spectra were acquired on two other samples: unlabeled stearic acid (2.5 wt %) in octadecane at 40 °C, as well as ^13^C-enriched stearic acid in 0.05 M aqueous NaOH at 70 °C. The latter sample was prepared by a melt blending method^41^ at 70 °C and the mass ratio of surfactant: oil is 1:8, which is identical to the one in the PCM nanoemulsion.

The ^13^C single-pulse NMR spectrum of stearic acid dissolved in octadecane at 40 °C exhibits a single ^13^C signal at 182.5 ppm, indicating the environment and corresponding ^13^C chemical shift of stearic acid solubilized in octadecane. For the uniformly ^13^C-labeled stearic acid in 0.05 M NaOH at 70 °C, two ^13^C signals appear as a consequence of ^13^C–^13^C *J*-coupling (^1^*J*_cc_ of 52 Hz). The ^13^C chemical shift of the carbonyl headgroup is 185.8 ppm, an increase of 2.3 ppm compared to stearic acid in octadecane. Here, stearic acid is deprotonated and predominantly in micelles; therefore, this ^13^C chemical shift is thus indicative of a deprotonated carbonyl group in aqueous media. Note that the typical single-bond ^13^C–^13^C *J*-coupling constant for a single bond is around 45 Hz, but increases due to electronegative substituents^42,43^ such as COO^–^. Indeed, we measured and resolved the ^13^C–^13^C *J*-coupling of the ^13^C signals associated with the aliphatic tail groups (Figure S7), including the expected *J*-coupling triplet associated with the carbon next to the surfactant headgroup (carbon “17”).

The ^13^C single-pulse NMR spectra of the ^13^C-enriched PCM nanoemulsions reveal that the surfactant head groups are present in distinct chemical environments at different temperatures, providing insights into the local environments and mobilities of the surfactant head groups. At 10 °C, octadecane is solid (Figure 2c,d). Thus, the ^13^C signal of the carbonyl group becomes broad and featureless, resulting in weak (but nonzero) ^13^C signal intensity spanning from 180 to 186 ppm. Interestingly, this result indicates that the mobility of the surfactant head groups is greatly restricted but not completely frozen. At 30 °C, where the octadecane has completely melted, a broad ^13^C carbonyl signal centered at 183.1 ppm is observed, associated with the deprotonated surfactant head groups at the oil–water interface. Interestingly, the broad ^13^C signal of the surfactant head groups establishes that their local molecular mobilities are not sufficiently rapid and/or isotropic at this temperature to completely average their ^1^H–^13^C dipole–dipole interactions, which are not averaged to zero. In addition, a lower intensity ^13^C signal at 181.8 ppm is observed at 30 °C that is narrow and exhibits resolved *J*-couplings (^1^*J*_cc_ = 52 Hz). The origin of this ^13^C signal is intriguing because its lower ^13^C chemical shift indicates that it is more shielded (greater electron density), suggesting that it may be due to protonated surfactant head groups present within the oil phase. An alternative explanation for the origin of these two ^13^C signals is the formation of hydrogen bonds between two adjacent surfactant head groups, which has been shown to result in two distinct ^13^C signals in other systems rich in carboxylic acid groups, such as poly(acrylic) acid.^44^^13^C longitudinal *T*_1_ relaxation time measurements indicate that the ^13^C signals at 183.1 and 181.8 ppm have ^13^C *T*_1_ times of 462 and 818 ms, respectively (Figure S8), further indicating that the surfactant head groups are in two different environments with different molecular mobilities. At 40 °C, the ^13^C signal at 181.8 ppm disappears, indicating that enhanced thermal motions enable a greater population of the stearic acid to move to the oil–water interface, which is its thermodynamically preferred state.

At 45 °C, a new ^13^C signal emerges at 183.7 ppm, which is narrow and exhibits ^13^C–^13^C *J*-couplings (^1^*J*_cc_ = 51 Hz), indicating increased molecular mobility. At 50 °C, the intensity of this narrow ^13^C signal increases, while the broader ^13^C signal at 183.1 ppm disappears as the molecular mobilities of surfactant headgroups continue to increase as the temperature increases. Thus, at 50 °C, the surfactant head groups are isotropically mobile. Due to the increase in mobility coupled with the increase in ^13^C chemical shift, we hypothesize that the surfactant head groups are, on average, more solvated by water molecules to a greater extent in the aqueous phase. In fact, the corresponding liquid-state ^13^C single-pulse NMR spectra of the aliphatic region (Figure 3b), which are dominated by octadecane but reveal unique ^13^C signals associated only with the stearic acid (“17” and ‘16', Figure 2b), reveal that the carbon moieties adjacent to the carbonyl headgroup also become increasingly mobile as the temperature is increased. In combination, the increasing ^13^C signal intensities of these carbon moieties (‘16', ‘17', and “18”) at higher temperatures, coupled with the increased ^13^C chemical shift of the carbonyl headgroup, suggest that the stearic acid headgroups become more solvated by water at the oil–water interface.

The total integrated ^13^C signal intensity of surfactant head groups as a function of temperature (Figure 3c) follows a similar trend to the total integrated ^1^H signal intensity of the aliphatic moieties (Figure 2d, predominantly octadecane). However, as noted above, the carbonyl groups do not completely freeze at lower temperatures, retaining significantly reduced and possibly anisotropic mobility at the oil–water interface below the freezing point of octadecane. A schematic of the molecular distributions and behavior of the surfactants at the oil–water interface as a function of temperature is proposed, as shown in Figure 3d.

### Mobility Loss of Surfactant Head Groups during Extended Thermal Cycling

**Figure 4:**
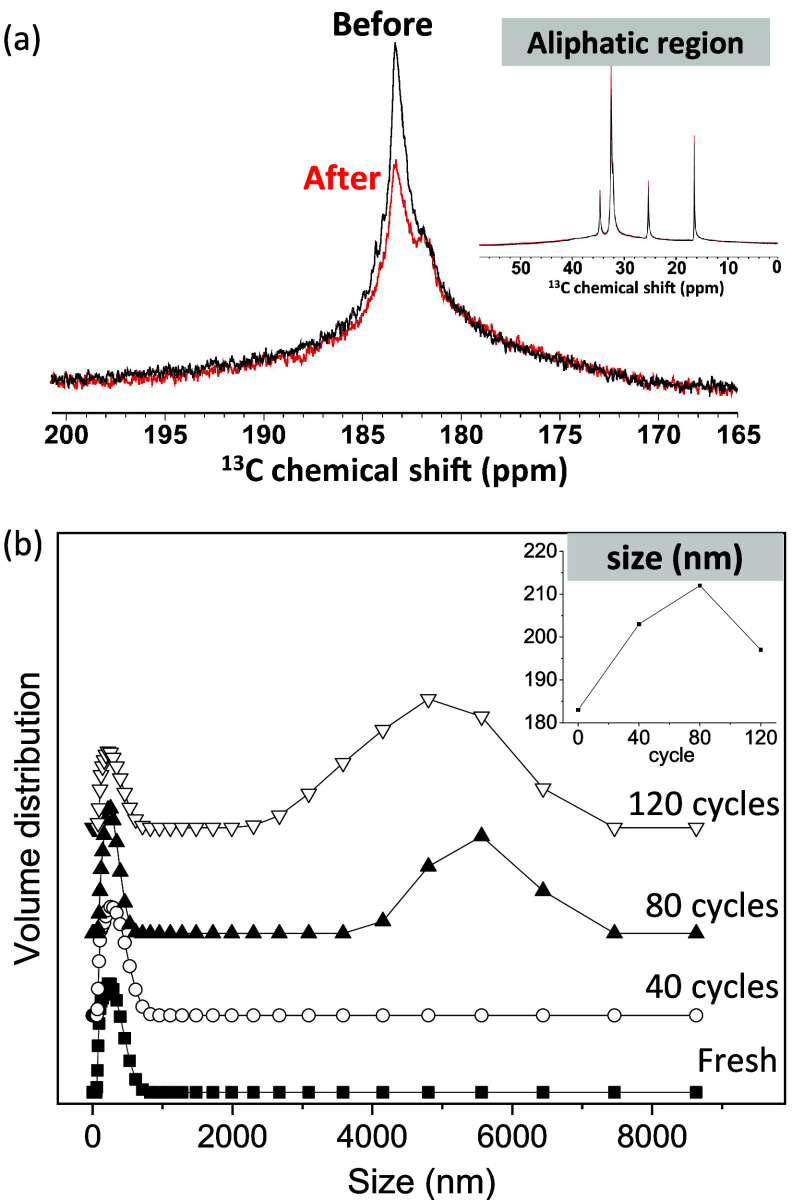
(a) Quantitative liquid-state ^13^C single-pulse NMR spectra of the stearic acid carbonyl headgroup of a PCM nanoemulsion (20 wt % octadecane, 2.5 wt % stearic acid (uniformly labeled), 77.5 wt % aqueous 0.05 M NaOH), acquired initially and after 120 thermal cycles at 40 °C. The integrated ^13^C signal intensity decreases by 22%, indicating the loss of molecular mobility. Inset: ^13^C single-pulse NMR spectra of the aliphatic tail groups. (b) Mean droplet size (inset) and distribution of a PCM nanoemulsion (20 wt % octadecane, 2.5 wt % unlabeled stearic acid, 77.5 wt % aqueous 0.05 M NaOH) upon repeated thermal cycling.

To understand the effect of repeated thermal cycling on the PCM nanoemulsion, we thermally cycled the sample within the NMR tube 120 times between 5 and 40 °C, acquiring quantitative ^13^C single-pulse NMR spectra upon thermal cycling. The ^13^C carbonyl surfactant headgroup signal before and after thermal cycling is shown in Figure 4a. It is interesting to note that the ^13^C carbonyl surfactant headgroup signal intensity decreases by 22% and the peak becomes broader and separates into two peaks while the aliphatic region signal intensity is almost unchanged (<1%, Figure 4a, inset). This decrease indicates that the molecular mobility of the surfactant headgroup was significantly reduced due to the melting and freezing process during repeated thermal cycling. The physical origin of this decrease in headgroup mobility is still under investigation; however, it indicates that less (mobile) surfactant stabilizes the oil–water interface, which would be expected to reduce the phase stability of the nanoemulsions upon repeated thermal cycling.

DLS measurements were performed on the PCM nanoemulsion every 40 cycles, which was thermally cycled in an oven 120 times between 5 and 40 °C (Figure 4b). We observed that the droplet size distribution becomes broader during thermal cycling and the appearance of the aggregation after applying 80 thermal cycles. In the inset figure, the mean droplet size, which includes the additional peak at larger sizes, increases and reaches the maximum after 80 thermal cycling and decreases after 120 thermal cycles showing that the creaming occurred after 80 thermal cycling and the aggregated emulsion droplets migrate upward thus resulting in the gradient of concentration of oil in the direction of height. Therefore, the mean droplet size decreases again after 120 thermal cycles, but it is still greater than one of the pristine samples. This result shows that the PCM nanoemulsion becomes unstable due to the aggregation of emulsion droplets during repeated thermal cycling. Therefore, the mobility loss of the surfactant headgroup is clearly linked to the aging or phase instability of PCM nanoemulsion due to repeated melting and freezing processes during thermal cycling.

### Understanding of Average Size Changes of PCM Nanoemulsion

**Figure 5:**
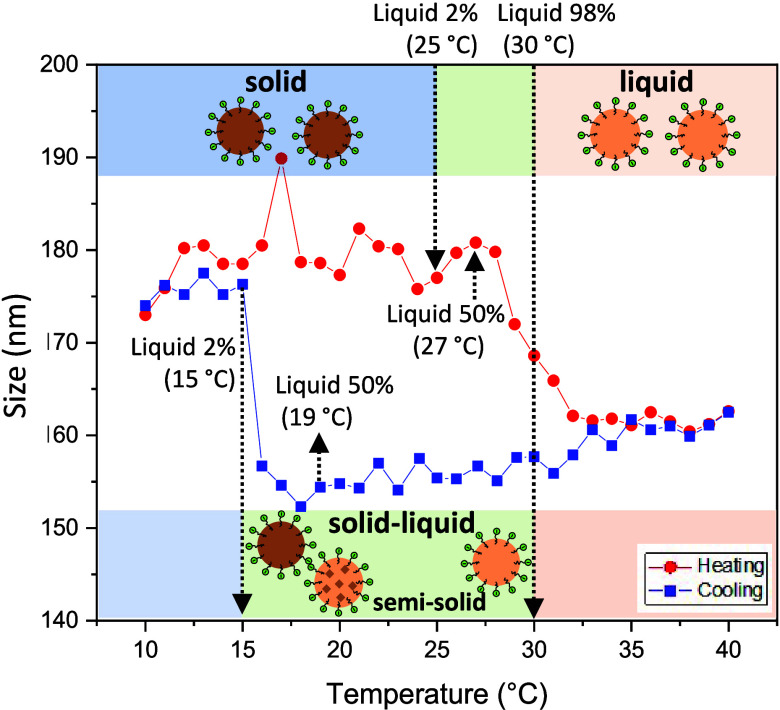
Mean droplet size of a PCM nanoemulsion measured by DLS during thermal cycling correlated with the liquid content of octadecane quantified by liquid-state ^1^H NMR measurements. The area was divided into three regions: (i) a solid region (blue), where the liquid content of oil was <2%, (ii) a liquid region (orange), where the liquid content was >98%; and (iii) a solid–liquid coexistent region (green) where the liquid content was in between 2 and 98%. The schematic images describe the state of PCM nanoemulsion droplets in each of these regions.

To better understand how the phase transition of octadecane affects the mean droplet size changes as a function of temperature, the mean droplet size was measured by DLS. The mean droplet size was also correlated (Figure 5) with the liquid fraction of the oil phase measured by NMR (Figure 2d). Based on the liquid fraction of the oil phase, we divided the range of the temperatures into three regions including a solid region, where the liquid content of the oil phase is less than 2%; a liquid region, where the liquid content of the oil phase is greater than 98%; and a solid–liquid coexistent region lying in between. Schematic images were used to describe the state of PCM nanoemulsion droplets in each of these regions.

The mean droplet size of a PCM nanoemulsion exhibits distinct trends during thermal cycling. It increases significantly from 15 to 17 °C, just before octadecane freezes (15 °C) where the liquid fraction of the oil phase is 2%. As the heating process continues, the droplet size fluctuates until it undergoes a significant decrease at 27 °C, where the liquid fraction of octadecane is 50%, corresponding to the onset of octadecane melting. While the sample was cooled, the mean droplet size gradually decreased until it reached 15 °C, after which it returned to its original average size. Furthermore, the droplet size distribution changes from monodisperse to bidisperse around the freezing and melting temperatures during both the heating and cooling processes (Figure S9). We observed that the solid–liquid coexistent region is significantly larger during the cooling process (∼15 °C window) compared to the heating process (∼5 °C), which shows the supercooling effect of octadecane. Both the size hysteresis and fluctuations upon thermal cycling are reproducible (Figure S10).

This result can be understood through the emulsion destabilization mechanism that can occur near the solid–liquid phase transition, as discussed in the literature.^45−47^ The coalescence of emulsion droplets occurs when semisolid droplets collide with each other or when solid particles and supercooled liquid droplets collide. During the phase transition of octadecane, the solid crystal of octadecane breaks the surfactant film, leading to coalescence, a phenomenon known as the “pin effect”.^46,47^ The solid particle acts as a nucleating agent in the supercooled liquid droplet, resulting in partial fusion between them, which can form a bigger droplet after the melting of octadecane. This process has been observed using optical microscopy by Golemanov et al.^45^ On the other hand, the collision between two solid particles will not result in coalescence, while the collision of two liquid droplets stabilized by surfactants at the oil–water interface may not result in coalescence. When solid particles collide, the contact area between the particles is often too small to provide a sufficient adhesion force, making them easily separable. Similarly, when liquid droplets collide, stearic and electrostatic repulsive forces associated with the surfactants at the interface prevent them from coalescing. This mechanism can account for the observed changes in the mean droplet size of a PCM nanoemulsion during thermal cycling and can explain why the mean droplet size increases and fluctuates in the solid–liquid coexistence region, followed by a decrease in the liquid region. Therefore, repeated freezing and melting processes during thermal cycling would lead to an overall increase in the mean droplet size resulting in coalescence which can eventually render the emulsion unstable or result in phase separation.

## Conclusions

A model PCM nanoemulsion was designed using octadecane as the oil phase, stearic acid as the surfactant, and aqueous NaOH as a continuous phase. The phase stability and droplet size distribution were monitored using DLS at different temperatures over time, where the droplet size distribution became broader and the mean size increased as the emulsion aged, leading to phase separation. Liquid-state ^1^H single-pulse experiments were performed during thermal cycling, enabling noninvasive quantitative measurements of the liquid fraction of the oil phase as a function of temperature. Octadecane melted abruptly above 26 °C, while complete melting was obtained at 30 °C. In contrast, the octadecane solidified gradually upon cooling, where approximately 50% of the oil phase was solid at 19 °C and complete freezing was observed at 15 °C. Liquid-state ^13^C single-pulse NMR measurements revealed that the surfactant head groups were present in multiple distinct environments throughout the melting process, suggesting their presence in both the aqueous and oil phases. Quantitative analysis of the ^13^C carbonyl signals reveals that the surfactant carbonyl head groups lose mobility during cooling, retaining significantly reduced and possibly anisotropic mobility at the oil–water interface below the freezing point of octadecane.

After 120 thermal cycles, the ^13^C surfactant carbonyl signal intensity decreased by 22%, indicating a loss of surfactant headgroup molecular mobility; in contrast, ^13^C aliphatic signals associated predominately with octadecane alkyl tails decreased by less than 1%. While the precise origin of this loss in surfactant headgroup mobility upon repeated thermal cycling is under investigation, it appears to be a precursor to phase instability. The mean droplet size of the PCM nanoemulsions, determined by DLS, was analyzed in relation to the liquid fraction of octadecane measured by liquid-state ^1^H single-pulse NMR spectroscopy. This analysis explains the changes in the mean droplet size during thermal cycling, resulting in an increase and fluctuation in droplet size within the solid–liquid coexistence region, which is associated with phase instability. Overall, liquid-state NMR spectroscopy is shown to be a powerful method for noninvasively quantifying the degree of supercooling in the organic PCM phase, as well as for probing the molecular environments and dynamics of surfactants at the oil–water interface. These results yield new insights into the mechanisms of aging and phase instability upon thermal cycling in PCM nanoemulsions.

## Supporting Information Available

Chemical structure of components and schematic image of model PCM nanoemulsion, physical properties of components, composition matrix of PCM nanoemulsions and the description of their phase stability, photographs of the PCM nanoemulsion at different storage condition, liquid-state 2D ^1^H{^13^C} HSQC NMR spectrum of stearic acid in deuterated benzene, liquid-state ^13^C single-pulse NMR spectra of a PCM nanoemulsion and integrated ^13^C signal intensity during thermal cycling, solid-state ^13^C{^1^H} CP-MAS NMR spectra of a PCM nanoemulsion upon heating, liquid-state ^13^C single-pulse NMR spectra of a PCM nanoemulsion at 70 °C, ^13^C *T*_1_ NMR relaxation times of surfactant head groups, size distribution of a PCM nanoemulsion measured by DLS during thermal cycling, and mean droplet size of a PCM nanoemulsion measured by DLS during thermal cycling (PDF)
