# Supplementary material for: Molecular-Level Understanding of Phase Stability in Phase-Change Nanoemulsions for Thermal Energy Storage by NMR Spectroscopy
Source: Langmuir. 2024 Sep 30;40(41):21814–23. doi: 10.1021/acs.langmuir.4c02997 (PMC11483738; doi:10.1021/acs.langmuir.4c02997)
Supplement: Supplementary file 1 — la4c02997_si_001.pdf [file la4c02997_si_001.pdf]

Supporting Information

Molecular-Level Understanding of Phase Stability in Phase-Change Nanoemulsions for Thermal Energy Storage by NMR spectroscopy

Jungeun Park[1], Ulrich Scheler[2], Robert J. Messinger[1,*]

[1]*Department of Chemical Engineering, The City College of New York, CUNY, New York, NY, 10031, USA*
[2]*Center for Multi-Scale Characterization, Leibniz-Institute für Polymerforschung Dresden e.V., 01069, Dresden, Germany*

*Email: rmessinger@ccny.cuny.edu

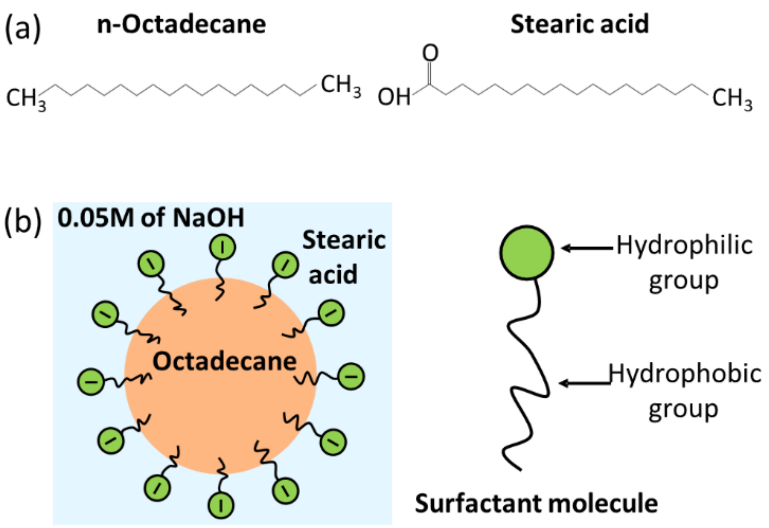

Figure S1. (a) Chemical structure of *n*-octadecane and stearic acid. (b) Schematic image of the model PCM nanoemulsion.

Table S1. Physical properties of octadecane and stearic acid.

	Octadecane	Stearic Acid
T_m (°C)	26-29	67-72
MW (g/mol)	254.49	284.48
Density (g/L)	0.777	0.941
Composition	$CH_3(CH_2)_{16}CH_3$	$CH_3(CH_2)_{16}COOH$

Table S2. Composition matrix of different nanoemulsions and comparisons of their phase stability and qualitative descriptions of their rheology at ambient temperature. Numerical values in the table represent mass percentages. "PS" denotes that phase separation occurred immediately after synthesizing the emulsion unless a different time is otherwise noted.

			**BC 1**	**BC 2**	**BC 3**	**BC 4**	**BC 5**	**BC 6**	**BC 7**	**BC 8**
Octadecane			0	20	20	20	20	20	20	20
Water			97.5	77.5	79.5	79	77.5	75	72.5	70
**Stearic acid**			2.5	2.5	0.5	1	2.5	5	7.5	10
total			100	100	100	100	100	100	100	100
**NaOH**	**1**	0 M		PS						
	**2**	0.01 M			PS		PS	PS		
	**3**	0.02 M			PS	PS	liquid to cream After 3 days			
	**4**	0.05 M	wax		hard surface lump inside	hard surface	**liquid milk PS after 8 weeks**	Sediment after 5 days		
	**5**	0.1 M				gel-like	gel-like	Soft gel-like PS after 2 weeks	heavy cream	
	**6**	0.2 M					hard gel	hard	sticky cream	heavy cream
	**7**	0.3 M						hard	hard	rubber
	**8**	0.5 M							very hard	solid
	**9**	0.7 M								solid

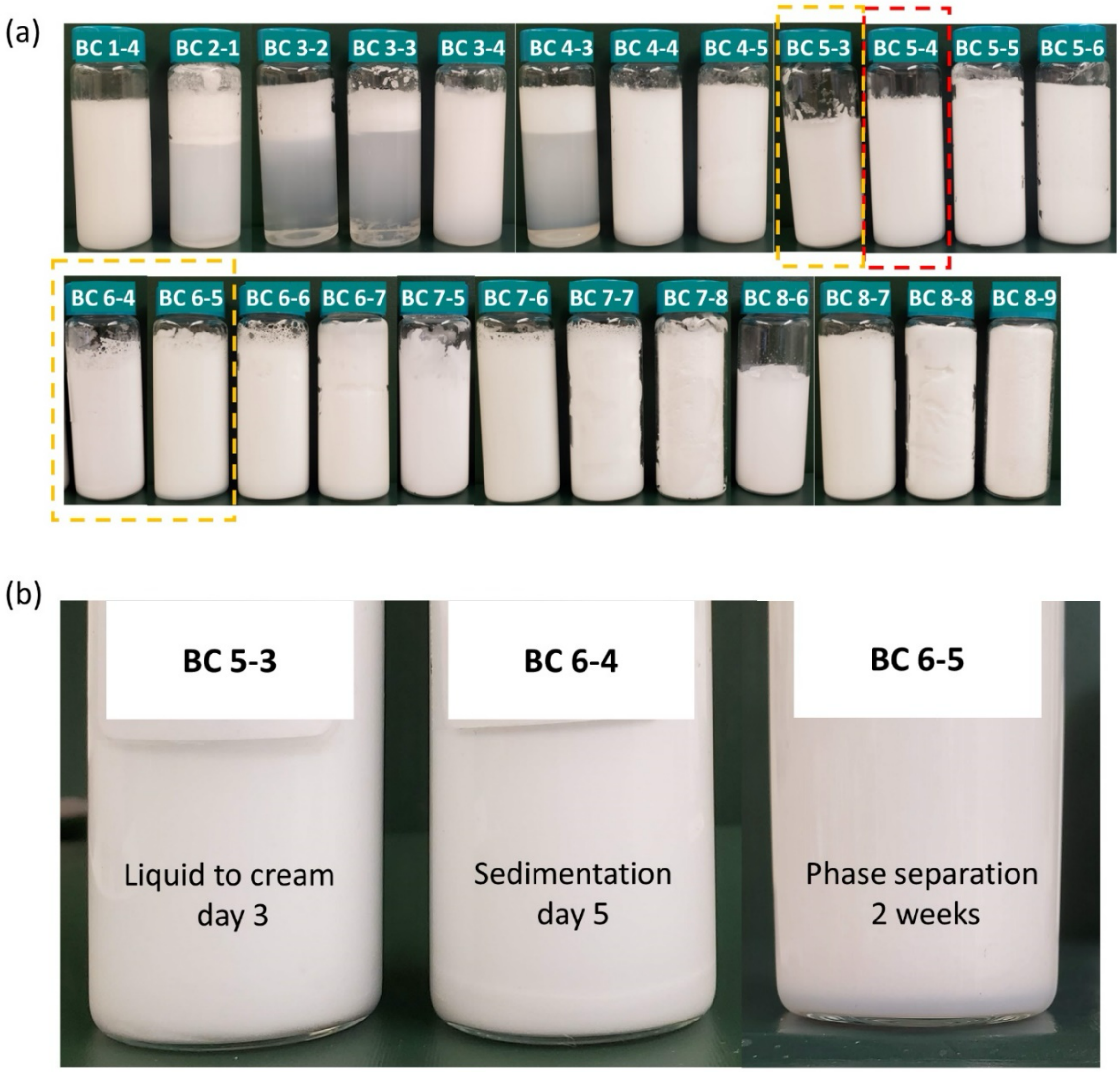

Figure S2. Photographs of the PCM nanoemulsion samples from the composition matrix (Table S2) (a) after 2 weeks stored at ambient temperature. (b) Magnified images of sample BC 5-3, BC 6-4, and BC 6-5. The number with dash increases as the concentration of NaOH increases in the composition matrix.

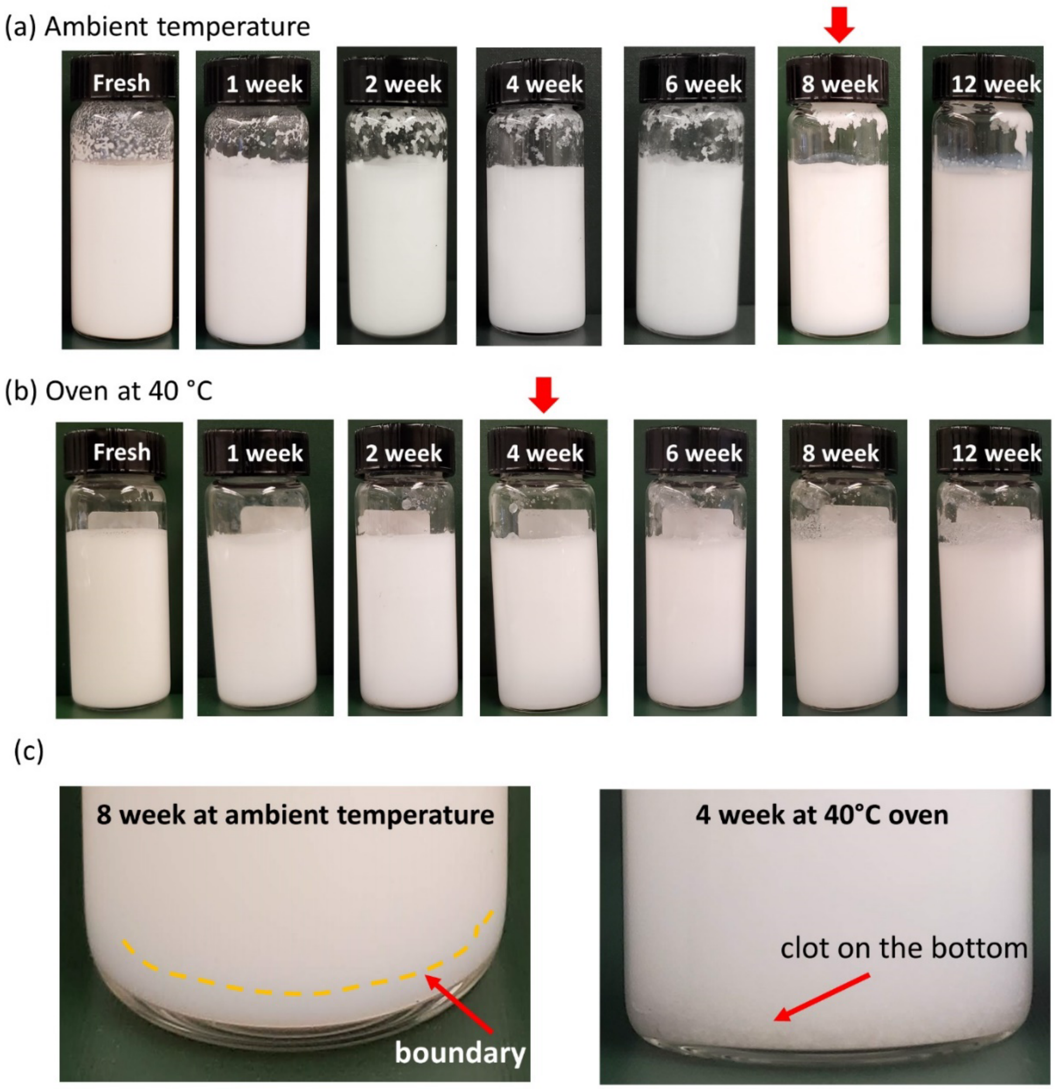

Figure S3. Photographs of the PCM emulsion stored at (a) ambient temperature and (b) in an oven at 40 °C. (c) Magnified images of samples showing a phase instability.

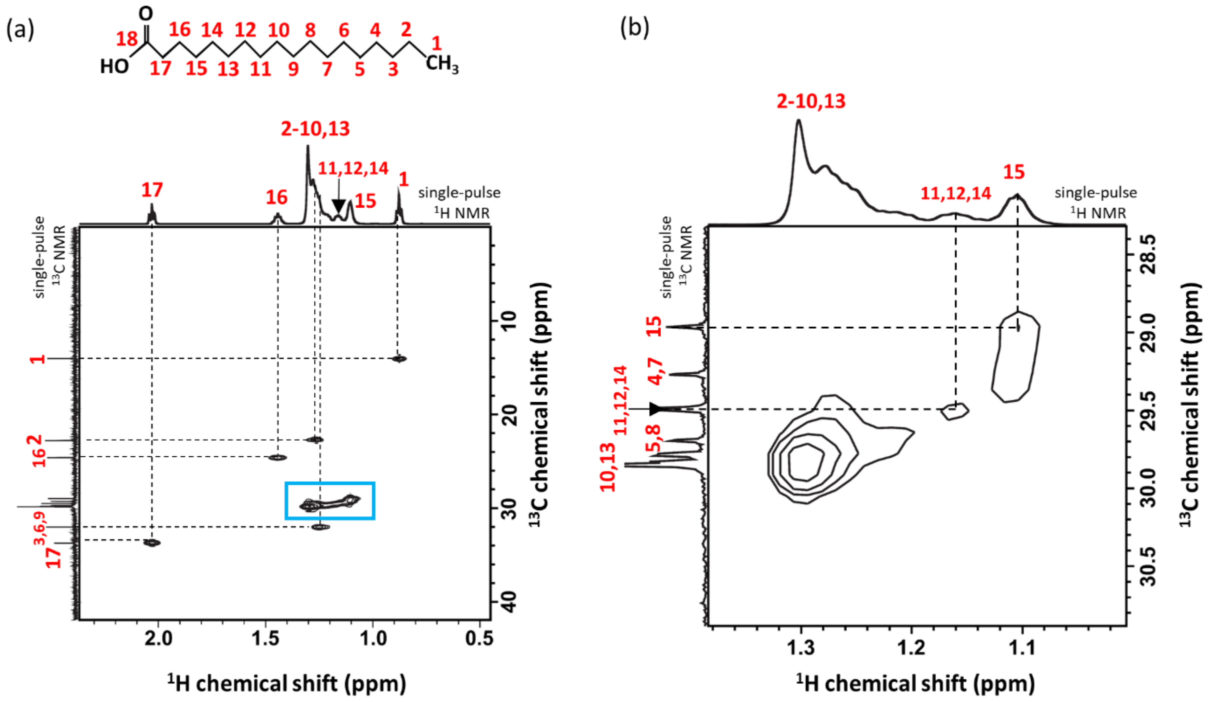

Figure S4. (a) Liquid-state 2D $^1H\{^{13}C\}$ HSQC (heteronuclear single quantum coherence) NMR spectrum and (b) magnified spectrum of the blue-colored square of stearic acid in deuterated benzene (0.01 wt%). Separately acquired ^1H and ^{13}C single-pulse NMR spectra are displayed along the horizontal and vertical axes, respectively. A schematic diagram of the stearic acid molecule is labeled with signal assignments for ^{13}C moieties and their covalently bonded protons. Note that the carboxylic acid hydroxyl proton is not observed because it is covalently bonded to the neighboring oxygen atom and the experiment is only sensitive to one-bond ^1H-^{13}C *J*-couplings.

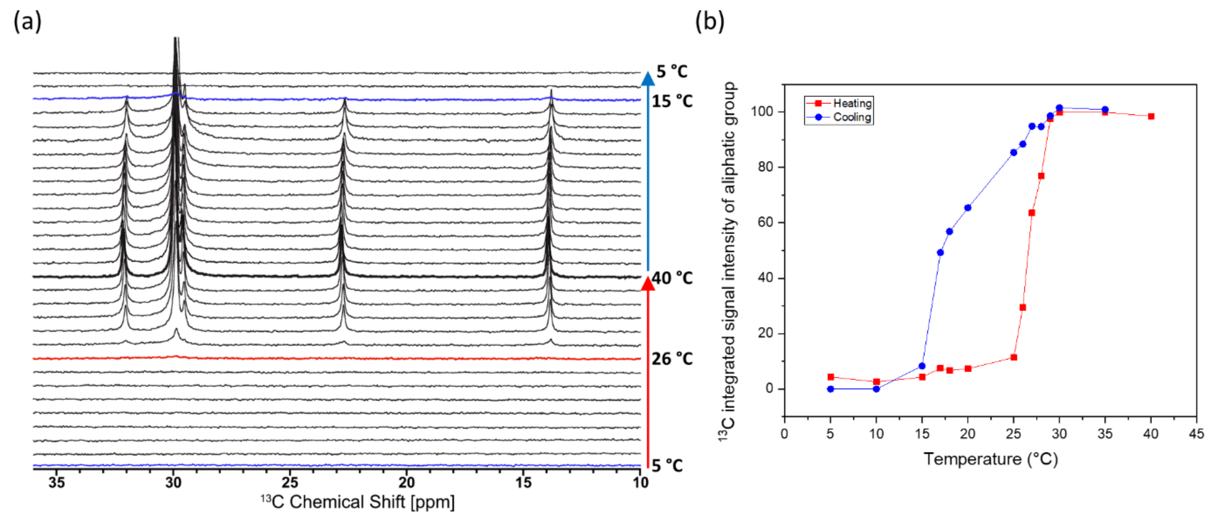

Figure S5. (a) Liquid-state ^{13}C single-pulse NMR spectra of a model PCM nanoemulsion (20 wt% octadecane, 2.5 wt% stearic acid, 77.5 wt% aqueous 0.05 M NaOH) during thermal cycling. (b) Integrated ^{13}C signal intensity of the aliphatic signals.

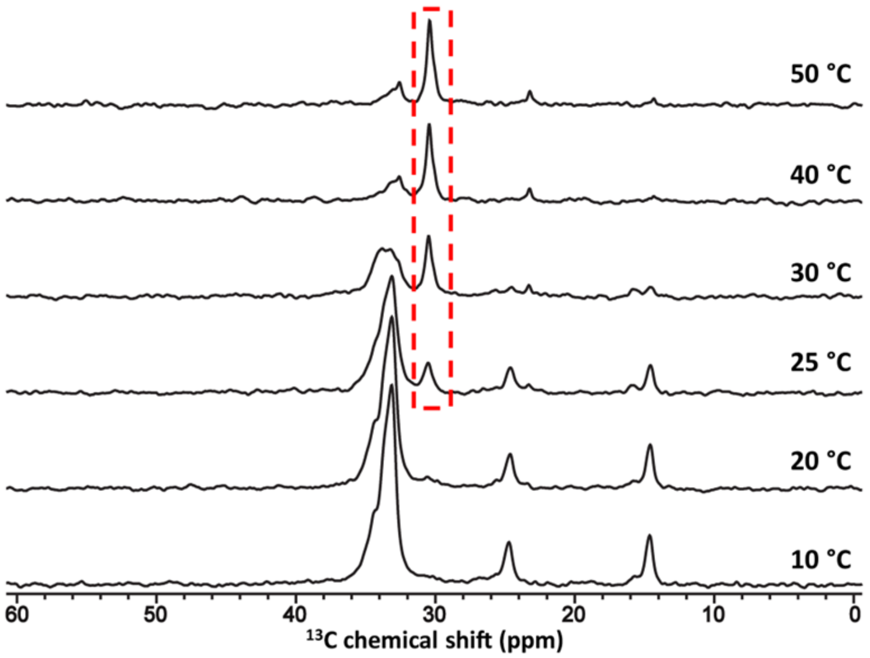

Figure S6. Solid-state $^{13}C\{^{1}H\}$ CP-MAS NMR spectra of the aliphatic region of a PCM nanoemulsion acquired from 10 to 50 °C. A new ^{13}C signal at 30.5 ppm appeared upon the melting of octadecane (red dashed box). This result suggests that a conformational change in the octadecane occurred, which restricted motion sufficiently to result in reduced motional averaging of the ^{13}C-^{1}H dipole-dipole coupling and the appearance of a $^{13}C\{^{1}H\}$ CP signal, even after melting. The NMR spectra were acquired at 10 kHz MAS and 14.1 T. Silicone spacers were placed between the samples and the end and drive caps of the 1.6-mm zirconia rotor, which upon sample rotation, sealed the sample with the rotor and prevented egress of any liquid.

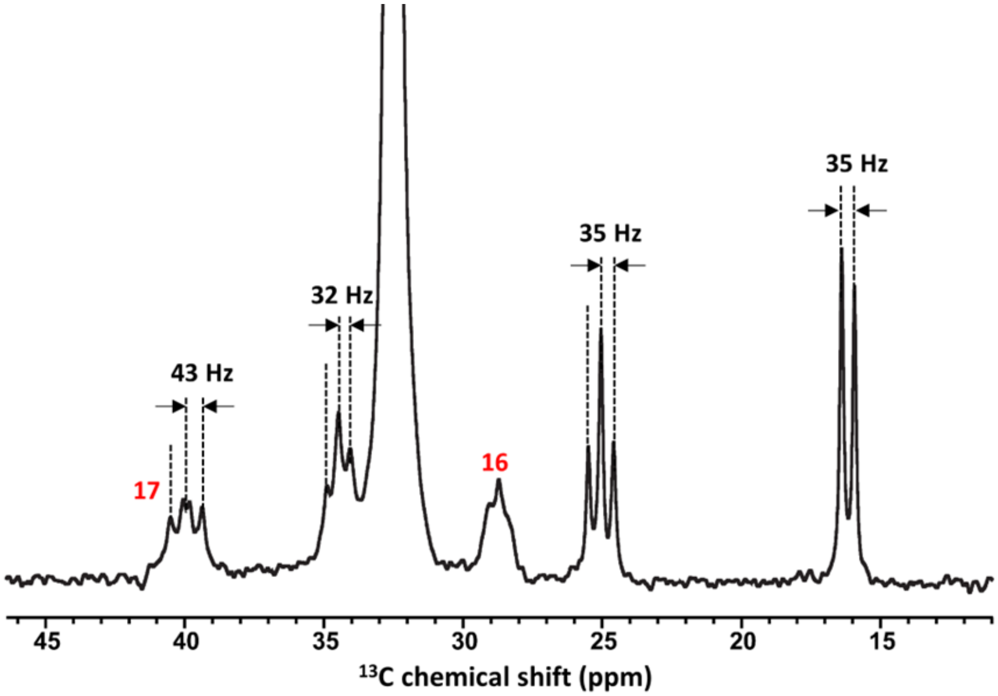

Figure S7. Quantitative liquid-state ^{13}C single-pulse NMR spectra of the aliphatic tail groups of uniformly ^{13}C-labeled stearic acid in 0.05 M aqueous NaOH measured at 70 °C. Notably, the ^{13}C-^{13}C J-coupling (43 Hz) of the carbon next to the surfactant head group (‘17’ in Figure 3b) is a triplet, as expected.

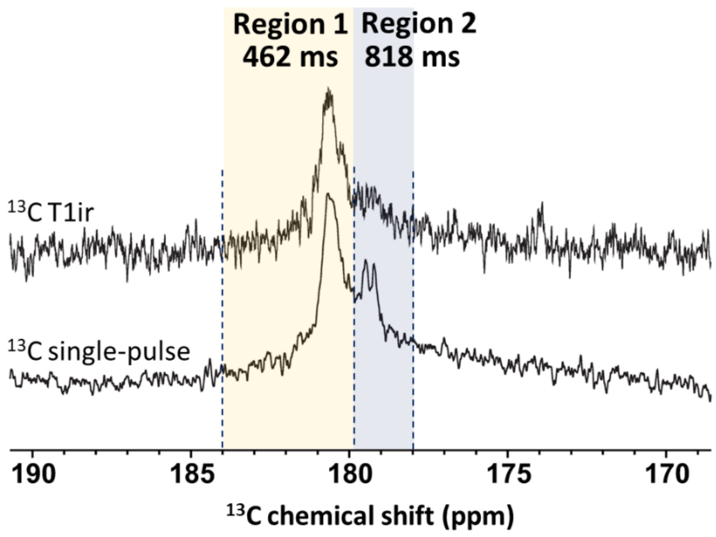

Figure S8. Quantitative liquid-state ^{13}C single-pulse NMR spectra and 1st slice from ^{13}C T_1 inversion recovery spectra of the model PCM nanoemulsion (20 wt% octadecane, 2.5 wt% stearic acid (uniformly labeled), 77.5 wt% aqueous 0.05 M NaOH), as well ^{13}C T_1 relaxation times, measured at 40 °C. The area from 179.8 to 184 ppm (region 1) and from 178 to 179.8 ppm (region 2) were integrated separately, yielding ^{13}C T_1 relaxation times of 462 ms and 818 ms, respectively.

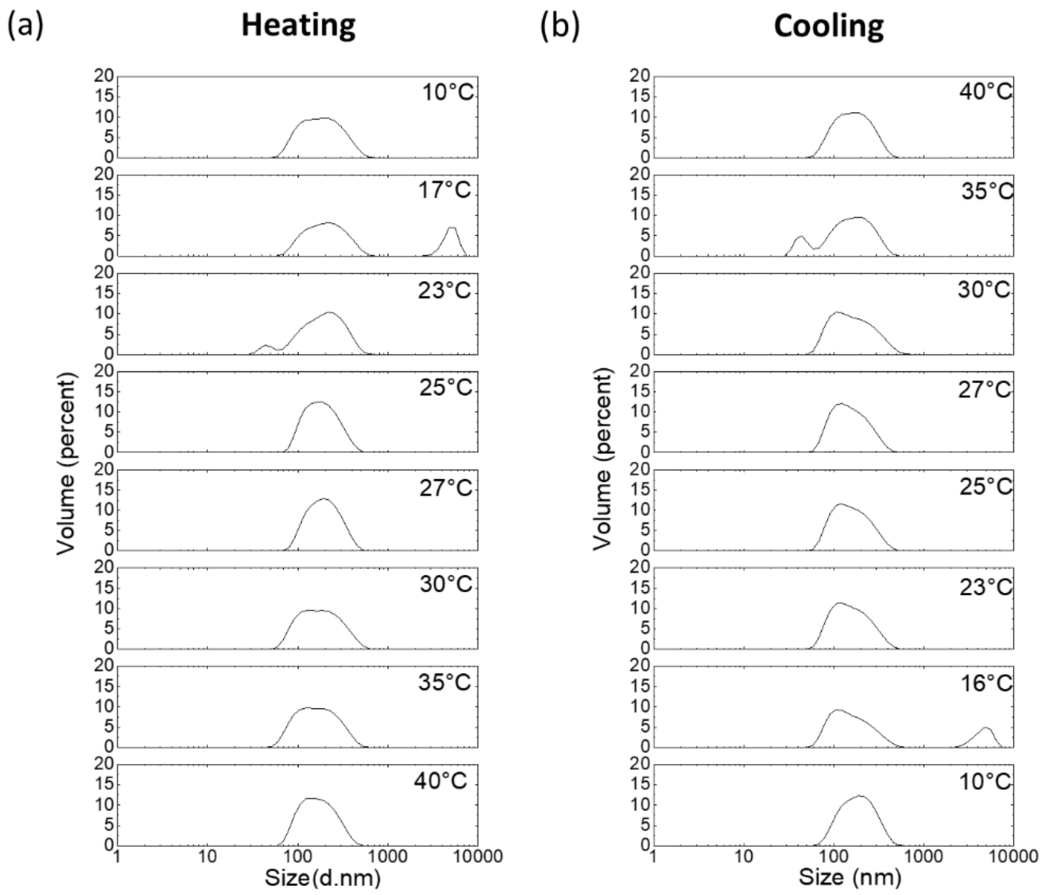

Figure S9. Volume-weighted droplet size distribution of a PCM nanoemulsions upon (a) heating and (b) cooling during thermal cycling as measured by dynamic light scattering (DLS). A refractive index of 1.468, 1.439, and 1.432 was used for solid octadecane below 15 °C, solid-liquid octadecane from 16 °C to 29 °C, and liquid octadecane above 30 °C, respectively.

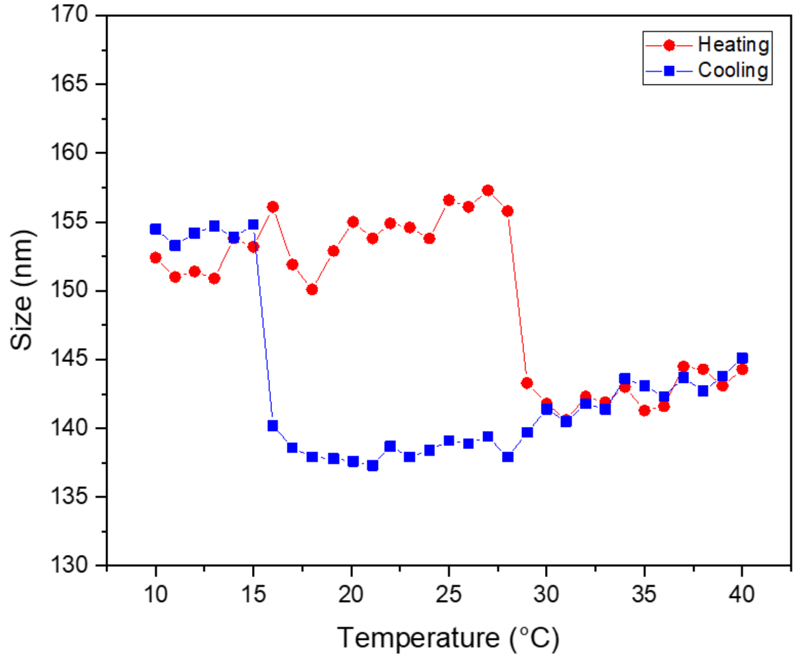

Figure S10. The mean droplet size of a PCM nanoemulsion measured by DLS during thermal cycling. The experiment is identical to the one reported in Figure 5 (main article) but was conducted on a different sample. While this sample had a slightly smaller average nanoemulsion size (by ~15-20 nm), both the size hysteresis and the fluctuations upon thermal cycling exhibited similar trends, highlighting the reproducibility of these phenomena.
